# Detecting benzodiazepine use through induced eye convergence inability with a smartphone app: a proof-of-concept study

**DOI:** 10.3389/fdgth.2025.1584716

**Published:** 2025-05-30

**Authors:** Kiki W. K. Kuijpers, Markku D. Hämäläinen, Andreas Zetterström, Maria Winkvist, Marieke Niesters, Monique van Velzen, Fred Nyberg, Albert Dahan, Karl Andersson

**Affiliations:** ^1^ Department of Anesthesiology, Leiden University Medical Center, Leiden, Netherlands; ^2^ Kontigo Care AB, Uppsala, Sweden; ^3^ Centre for Human Drug Research, Leiden, Netherlands; ^4^ Department of Pharmaceutical Biosciences, Uppsala University, Uppsala, Sweden; ^5^ MediD Consultancy Group, Amsterdam, Netherlands; ^6^ Outcomes Research Consortium, Houston, TX, United States; ^7^ Skillsta Teknik Design och Kvalitet AB, Vänge, Sweden; ^8^ DHEAR, Skövde University, Skövde, Sweden

**Keywords:** substance use disorder, pupillometry, eye convergence, benzodiazepines, smartphone

## Abstract

**Background:**

Benzodiazepines (BZDs) are readily available potent drugs that act as central depressants. These drugs are widely used, misused, and abused. For patients with BZD use disorder, the traditional sobriety monitoring method is periodic urine tests.

**Methods:**

The utility of eye-scanning data related to non-convergence (the ability to cross eyes) collected using smartphones with the Previct Drugs app before and after ingestion of the BZD lorazepam for detecting BZD-driven effects was evaluated using data from 12 individuals from a historic clinical study (NCT05731999). Using a novel metric that represents the change in distance between irises when converging eyes, either in absolute terms (NCdiff) or individualized (NCdiffInd), classifiers were built using logistic regression.

**Results:**

The ability to converge eyes is a strongly individual and acquired skill that is impaired after ingesting lorazepam. The maximum NCdiff for a BZD-sober individual may be smaller than the impaired NCdiff for another individual. Using the NCdiff measured in a sober condition after approximately 1 week of regular eye-scanning as the individual baseline to form NCdiffInd produced a highly functional classifier with an area under the curve (AUC) = 0.88, which was superior to a classifier based on NCdiff with an AUC = 0.79.

**Conclusions:**

The loss of eye convergence induced by lorazepam is continuous, individual, and can be partial. Smartphone-based eye-scanning technology combined with a classifier adapted to the ability of eye convergence of individuals shows promising performance in detecting ingestion of lorazepam.

## 1 Introduction

Drugs of the benzodiazepine (BZD) family are used for many conditions, including anxiety, insomnia, muscle relaxation, spasticity relief (from central nervous system pathology), and epilepsy (1). As a readily available potent central depressant family of drugs, widespread use (as prescribed) (2), misuse (using more than prescribed) (3), and abuse (qualifying as substance use disorder) of benzodiazepines has become a significant public health dilemma with 0.3%–2% of the population being affected (4, 5). The frequent co-abuse of BZD with alcohol (6) and other substances (5, 6) is a major concern (7). The most serious example is the often-lethal effect of combining opioids with BZDs (8). Furthermore, withdrawal from BZD abuse (9) can cause severe problems with a risk of suicide for subjects with psychiatric comorbidities (10). In addition to the suffering of individuals struggling with substance abuse and their families, society is impacted by increased risk of traffic accidents (11, 12) and the costly use of healthcare resources (13).

Traditional sobriety monitoring methods, including periodic urine tests and clinical assessments (14), are expensive and require the patient to visit a clinic in person (15, 16), resulting in infrequent monitoring of patient sobriety during therapy that jeopardizes the therapeutic alliance (17). The imperative to detect and manage substance use in real-time drives the quest for more accessible, cost-effective, and patient-centered intensive outpatient solutions (18).

For decades, manual techniques for detecting substance effects through a police officer's ocular inspection of the eyes of a person suspected to be under the influence, including nystagmus (NY) and non-convergence (NC), have been used as documented in police handbooks and standardized field sobriety testing (SFST) protocols (19). However, detection of the use of therapeutic BZD has failed using the SFST eye tests based on pupil size and nystagmus (20). The incorporation of sophisticated eye movement analyses, such as smooth pursuit (21), impaired near point eye convergence (22), and eyelid fluctuation (23), marks a pivotal development in the field. Smooth pursuit involves the eye's ability to actively follow a moving object smoothly, a function that can be disrupted by the intake of central nervous system depressants such as alcohol or BZD (21). Intake of lorazepam and other BZD drugs has been shown to impair near point eye convergence, i.e., the active ability to cross one's eyes (22, 24, 25). Eyelid fluctuations, however, refer to involuntary eyelid movements that can also be indicative of BZD intake (23). Manual methods, as conducted in SFST, are, however, prone to subjectiveness and have poor repeatability, limiting their utility for general, routine remote monitoring.

Smartphones, with their advanced sensors and widespread use, present an excellent opportunity to develop new monitoring methods. The idea of using smartphones to monitor eye movements, such as NC, NY, and pupillary light reflex (PLR), for drug use detection is particularly promising (26). Conventional pupillometry, which measures the PLR, fails to adequately detect BZD-induced changes, pointing to the necessity for alternative or supplementary biomarkers (27, 28). Yet, how these metrics can be effectively applied in a smartphone format for monitoring BZD abuse remains largely unexplored.

This study aimed to bridge this gap by evaluating the effectiveness of a new smartphone application (29) designed for the self-administered monitoring of eye movements to detect BZD use. We introduce an innovative method for measuring eye convergence utilizing a mobile app, wherein the user's eye movements are captured via the smartphone's camera. The captured video data are subsequently analyzed through edge computing and artificial intelligence (AI) models to generate a novel metric, i.e., non-convergence represented by the difference in distance between irises when eyes look straight and when converged (NCdiff). NCdiff offers a continuous scale of non-convergence, aiming to surpass the limitations of traditional pupillometry and thus establishing itself as a reliable indicator of being under the influence of BZDs. Acknowledging the significant variability in baseline eye movement metrics among individuals and the need for initial training and testing to establish a stable baseline, we advocate for the individualized adjustment of the NCdiff measure. The study aims to evaluate the effectiveness of detecting BZD use with a smartphone by individualizing the NCdiff signal, providing NCdiffInd.

## 2 Materials and methods

### 2.1 Study design and ethics

The study (26) had an explorative, randomized, parallel open-label feasibility design that took place in a single center in the Netherlands. The study was conducted at the Anesthesia and Pain Research Unit at Leiden University Medical Center between February and July 2023. The study protocol was approved by the ethics committee METC-LDD (Leiden, the Netherlands; approval date 2 February 2023). All the study procedures were performed according to good clinical practice guidelines and adhered to the tenets of the Declaration of Helsinki. The study was registered in the public trial register clinicaltrials.gov with identifier NCT05731999. Prior to study enrollment, all subjects provided written informed consent. Adverse events were collected between study enrollment and the end of the study for each subject and were assessed based on severity and relatedness to study procedures.

The study design, the participants, and the data collection procedures are described in detail in Kuijpers et al. (29) and in the public trial register. In brief, eye characteristic data were collected with an app embedded in the Previct platform (version 2.18, Previct Drugs; Kontigo Care AB, Uppsala, Sweden) in parallel with frequent blood sampling to obtain reference concentrations (29). After an initial visit (visit 1) with brief training of the subjects in how to use the Previct Drugs App, video-recordings of eye characteristics were made by the participants themselves both at home and during the second visit when study drugs were administered (visit 2). In total, 48 healthy volunteers, aged 18–70 years, mixed male and female, with a body mass index of 18.5–30 kg/m^2^ and weight of 50–100 kg were included as previously described (29). Only data from the 12 subjects receiving the BZD lorazepam (2.0 mg Lorazepam Aurobindo, oral, Aurobindo Pharma, East Windsor, NJ, USA) are analyzed in this report. The number of subjects was selected to allow confirmation of historic findings by others (22, 24, 25).

At visit 2, the subjects arrived at the research unit after fasting for at least 6 h. Visit 2 started with the collection of data in a sober state and continued with eye characteristic data collection every hour until 5 h after drug intake. Eye characteristic data measurements during visits 1 and 2 were collected in two controlled light conditions, approximately 50 and 500 lux, corresponding to dim indoor lights and bright indoor lights, respectively. Light conditions were pre-installed in a dedicated no-daylight room using smart controllable lighting equipment (IKEA, Älmhult, Sweden) and validated using a luminometer (Sekonic Flashmate L-308, Sekonic Inc., North White Plains, NY, USA). The main purpose of including different light conditions was to evaluate the influence of ambient light on the PLR results, as previously described (29).

Blood samples were drawn from an intravenous access line at six time points in conjunction with eye testing to allow for pharmacokinetic analyses (Ardena Bioanalysis, Assen, the Netherlands).

The Previct Drugs App was programmed to collect data from three different eye-scanning procedures: PLR, NC, and horizontal NY (29). However, only data from NC are analyzed in this publication. NC data were collected using the front camera with the help of digitized voice guidance. The test was self-administered, i.e., the subjects conducted the test themselves.

### 2.2 Data analysis

**Figure 1:**
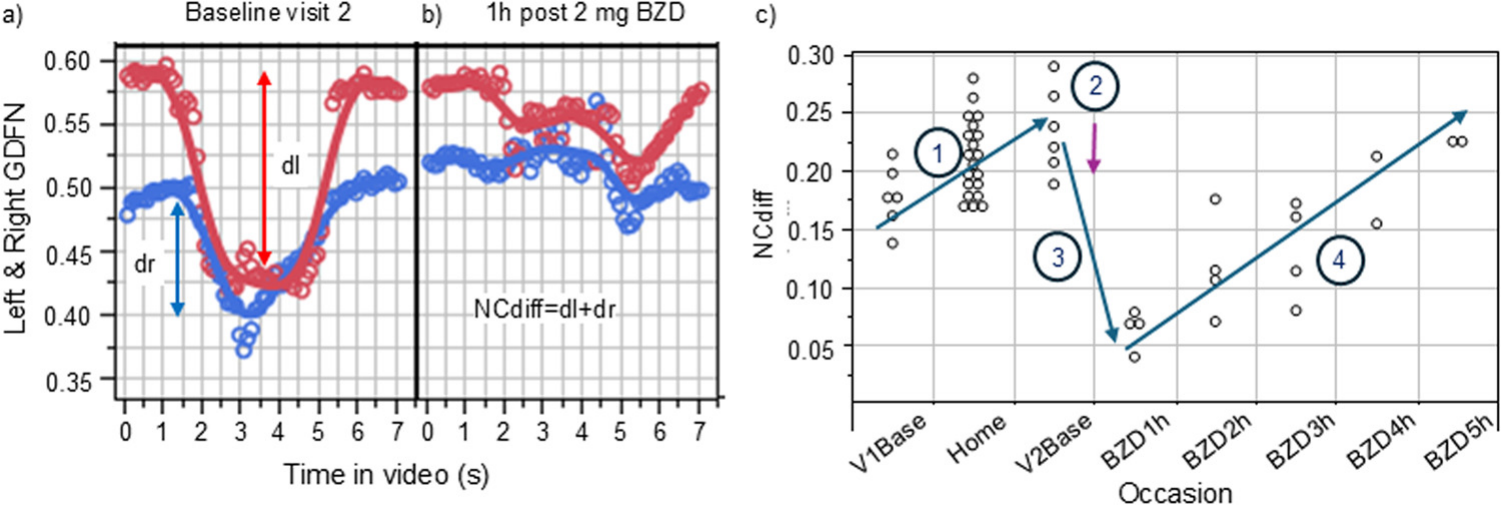
Illustration of the measured ability to converge one’s eyes (left eye, red, right eye blue) in a sober state **(a)** and under the influence of 2 mg of orally administered lorazepam. **(b)** The subject was video-filmed for 8 s, during which audio guidance told the subject to cross their eyes (at ∼1.5 s) and later to look straight ahead (∼5 s). GDFN is the gaze distance from the nose. NCdiff values from each measurement made during the study (first visit sober baseline, measurements at home, second visit baseline, and finally measurements under the influence of the BZD drug lorazepam) for subject B is shown in **(c)**.

The process of measuring eye convergence with the application involves several detailed steps, encompassing a quantitative assessment of the face, eye, iris, and the position of the iris relative to the nose for both eyes. The measurement procedure includes audio guidance that first helps the user find adequate lighting conditions and then guides the user to hold the phone in the correct way. It finally gives the instruction to “look straight,” approximately 1.5 s later gives the instruction to “cross your eyes,” and, finally, after approximately 5's, instructs the user to “look straight” again. Based on the 7.4-s-long video collected during the provision of instructions, the sum of the iris locations (calculated from the nose) obtained for the two conditions (look straight, cross your eyes) is estimated and subtracted, and denoted NCdiff. NCdiff is the sum of the left and right eye distances to the nose before the “look straight” and “cross your eyes” instructions (Figure 1). The obtained NCdiff values were, for each subject, normalized by subtracting the average sober baseline results at visit 2 (V2base) from all the collected data, denoted NCdiffInd. In this way, the effect of each drug is expressed as a deviation from one's own baseline. Descriptive statistics and regression modeling were conducted using JMP-Pro 16.2.0 -statistical software (SAS Institute Inc., Cary, NC, USA).

## 3 Results

The technique to measure change in the ability to converge eyes is illustrated in Figure 1a for baseline measurements at visit 2 and after the administration of the BZD lorazepam in Figure 1b. The graph in Figure 1a shows the gaze for each eye during the course of the 7.4-s-long video, calculated as the eye-to-nose distance. Figure 1b shows the results for the same subject conducting the same type of measurement when under the influence of lorazepam. As evident from the collected distances from nose to irises before and after the audio instruction to cross eyes (at ∼1.5 s), the ability to cross eyes was impaired. Figure 1c shows the NCdiff values from each measurement made during the study for subject B, starting with the initial training at visit 1 (V1base), followed by use at home (home), and thereafter the results from the sober baseline test on the day of administering lorazepam (V2base) and the results collected 1–5 h after administration of lorazepam. In this case, the subject had an increased ability to cross eyes during the use at home (Section 1). Such an acquired ability to converge eyes at home was seen in 5 of the 12 subjects in the study. The administration of lorazepam (Section 2) reduced their ability to cross eyes (Section 3) during the first 1–3 h when under the influence of lorazepam, followed by recovery (Section 4).

**Figure 2:**
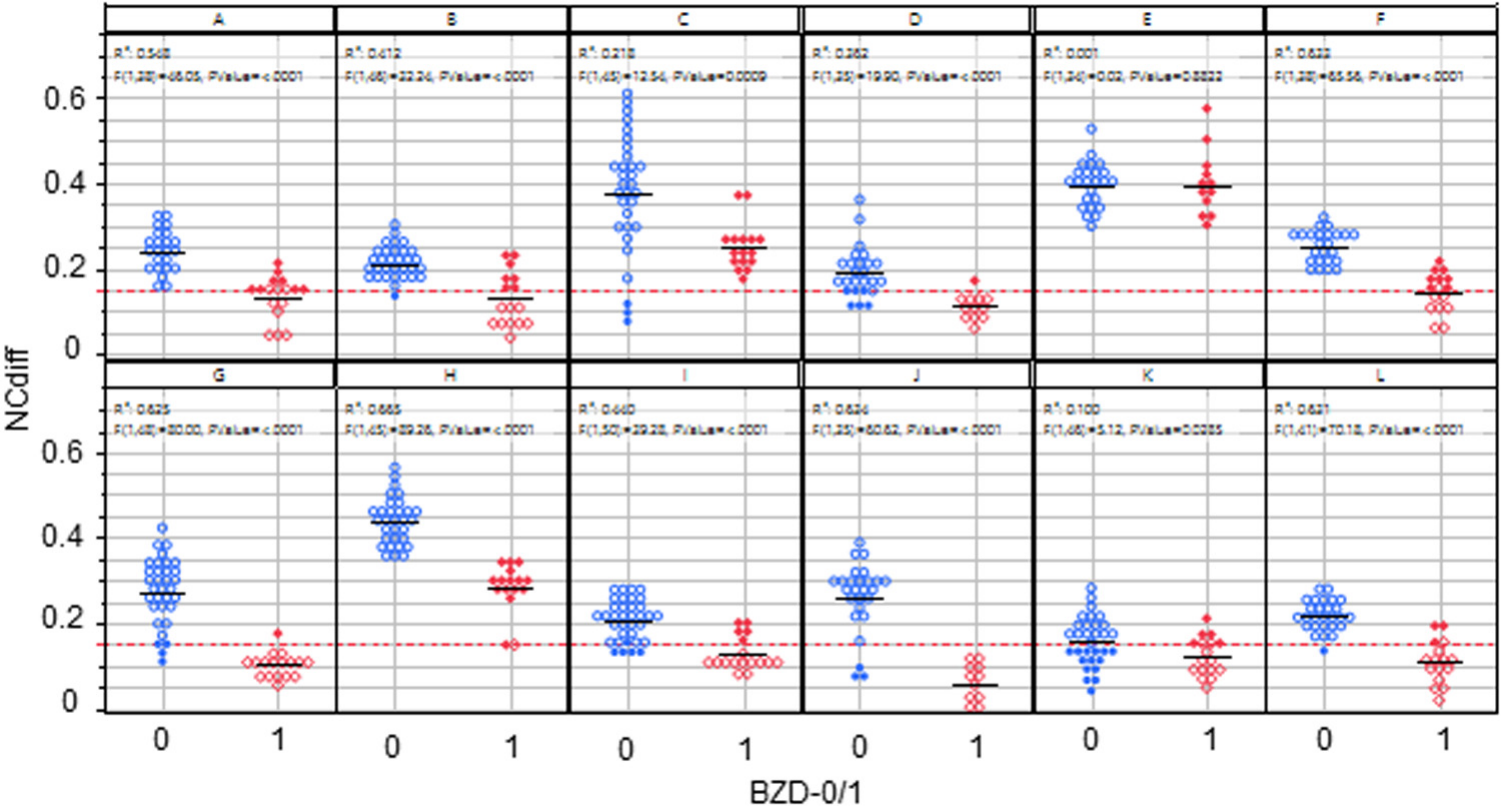
NCdiff results from all 12 subjects pre (BZD = 0) and post (BZD = 1) lorazepam administration. The red line indicates the classification limit. Open blue circles denote true negative results, filled blue circles denote false positive results, open red diamonds denote true positive results, and filled red diamonds represent false negative results.

**Figure 3:**
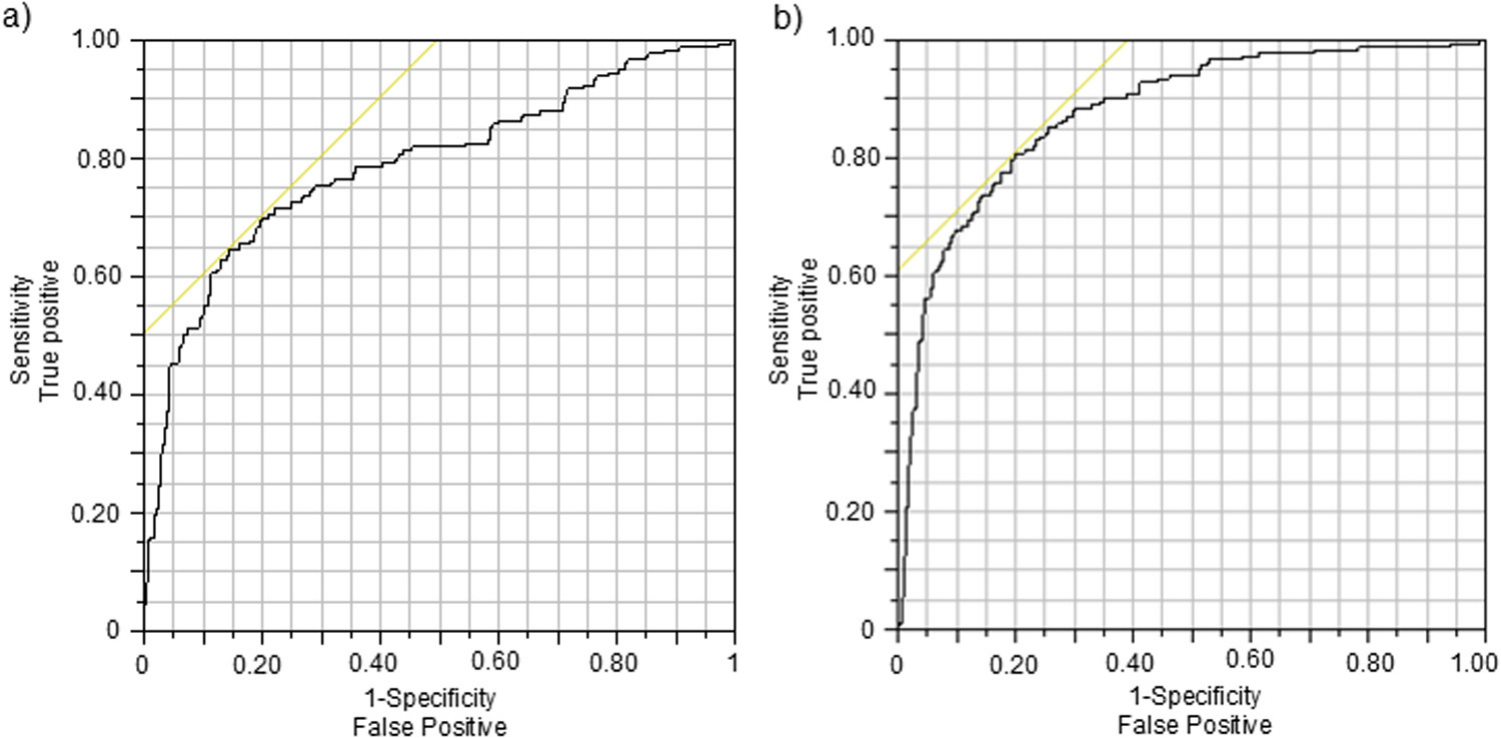
Receiver operator characteristic (ROC) graphs for classifiers indicating BZD intake based on **(a)** NCdiff and **(b)** NCdiffInd. The intersection of the yellow line and the black ROC curve defines the threshold for the classifier.

Figure 2 shows the NCdiff results from all 12 subjects pre (BZD = 0) and post (BZD = 1) lorazepam administration. The within-subject NCdiff values were significantly smaller after lorazepam administration in 11 subjects (*p* < 0.0009 for 10 subjects, *p* < 0.03 for 1) and insignificant for one of the 12 subjects. Subject E showed a good ability to cross eyes and no difference in NCdiff pre/post-lorazepam. The difference was also small for K, albeit significant (*p* < 0.03). The post-lorazepam result for subject C overlapped with the average baseline for four subjects (A, F, G, and J) and was even higher for five other subjects (B, D, I, K, and L), illustrating the need for individualized models of the ability to converge eyes for optimal indication of drug use. When using NCdiff alone as *x* to predict a target variable reflecting drug ingestion (pre/post-lorazepam administration = 0/1, *n* = 525, meaning each NCdiff measurement was evaluated independently) using logistic regression, the threshold for separating sober from under the influence of lorazepam was 0.15, as indicated by the red horizontal line in Figure 2. The logistic regression model had an area under the curve (AUC) value of 0.79 (Figure 3a) and a true negative (TN) rate of 0.89 and a true positive (TP) rate of 0.56, indicating that the effect size was large.

**Figure 4:**
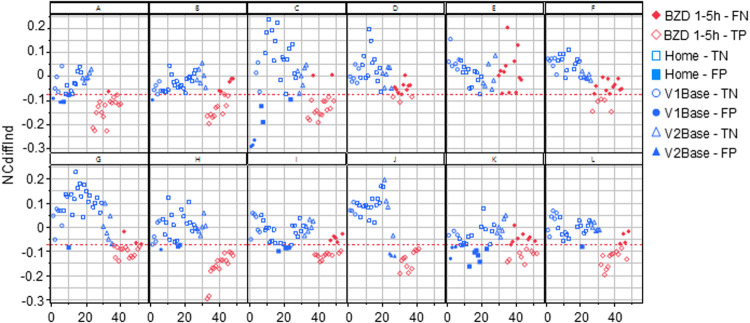
All the NCdiffInd results from all study subjects in the study are plotted in order of the performed tests. The red line indicates the classification limit. The open symbols represent true positives and negatives, while the filled symbols indicate false positives and negatives. V1Base and V2Base refer to the tests performed during the two hospital visits before drug administration. The Home-true negative (TN) / false positive (FP) tests represent all tests conducted at home between these visits. The BZD1-5h-false negative (FN) / true positive (TP) tests refer to those performed 1–5 h after the administration of 2 mg of lorazepam at visit 2.

To individualize the NCdiff measurements, the average baseline NCdiff value of each subject at their second visit (NCdiff @V2base) was subtracted from their other NCdiff values, resulting in the NCdiffInd values. The impact of this individualization was assessed by constructing logistic regression models on the entire dataset (*n* = 525), using the same target variable as previously. The model utilizing NCdiffInd alone achieved an AUC value of 0.88 (Figure 3b), with a TP rate of 0.67 and a TN rate of 0.90. The classification threshold for NCdiffInd was −0.075 (indicated by a dashed line in Figure 4). A considerable part of the false negative results (Figure 4, filled red diamonds) was related to the subjects who did not show a decreased NCdiff after lorazepam intake (E and K; Figures 2, 4). Subject K also contributed to nearly half of the false positives (Figure 4, filled blue circles, squares, and rectangles). Another subject with false negatives was D, who has corneal arcus and altered irises. Data collected 4–5 h after drug administration were false negatives for some subjects (B, G, and L). The presence of false positives at the start of the test series further indicates an acquired test skill effect, emphasizing the importance of adaptation over time. The NC-tests performed at home are primarily classified as TN (Figure 4, open blue squares) and were, in most cases, well-separated from the tests performed post-intake of lorazepam. Of the total 199 home tests, 179 were TN and 20 false positive (FP) (of which 7 belonged to subject K).

Before drug administration (blue symbols), there were trends of increased (A, B, C, H, and K; positive slope) and decreased (E and F; negative slope) ability to converge eyes over time (Figure 4, blue circles, squares, and rectangles). For a few subjects, the ability to converge eyes varied considerably over time. After drug administration, for six subjects, the immediate loss of ability to converge eyes slowly recovered, seen as a significantly positive NCdiffInd vs. test order slope (as exemplified in Figure 1 and seen in Figure 4 for subjects A, B, C, H, I, J, and L).

## 4 Discussion

Benzodiazepine drugs are widely used and abused in many regions of the world. Current monitoring methods are limited and include the analysis of body fluids such as blood, urine, or saliva. In this report, the correlation of eye reaction impairment to the intake of lorazepam was evaluated using a smartphone-based app, putatively allowing distributed follow-up of individuals using (or misusing) benzodiazepines. The significant differences in eye convergence observed between the drug-free home tests and the post-lorazepam intake measurements are in line with police training material (24) and highlight the potential of using a smartphone app to monitor pharmacological effects of benzodiazepines in an at-home setting. Since the subjects in the study were only instructed to perform the tests indoors when using the app at home, the collected data represents a fair average of the indoor environments encountered in and around the urban area of the city of Leiden (in the Netherlands).

The original analysis of the clinical study data as stipulated in the study protocol (26) was based on averaged data from visit 2 only, where it was shown that a decreased ability to perform NC was significantly correlated with the lorazepam drug effect at peak plasma concentration and the effect was significant up to 5 h after drug administration (29), confirming the impact of BZDs on convergence suggested by others (24, 25). This report extends the analysis of the relationship of BZD and NC by also including the data from the tests performed at home in the week before drug intake.

Furthermore, police handbooks (24) generally state that the ability to converge the eyes is something one can or cannot do. This study shows that the ability to converge the eyes and the extent to which this capacity is lost due to ingestion of a benzodiazepine drug is a quantitative value that is highly specific to an individual. As evident from Figure 2, there is notable individual variability in the baseline ability to perform eye convergence, highlighting the complexity of interpreting eye movement data. The administration of lorazepam resulted in a discernible but variable effect on the ability of the subjects to converge their eyes, with most experiencing a diminished capacity. This effect varied among the subjects and over time, showing the complex influence of lorazepam on eye function. Hence, the loss of eye convergence induced by lorazepam is continuous, individual, and can be partial. It is essential to embrace these characteristics when engineering an individualized classifier to estimate lorazepam intake.

We argue that the ability to converge the eyes is an acquired skill. During the eye tests conducted at home the week prior to the day of the intervention, some of the subjects improved their ability to cross eyes significantly (Figure 4). It has been discussed that 7.5% (range 1.7%–30%) of the population lack the ability to converge their eyes (30). We argue that the large variation in the reported prevalence of convergence inability may be due to variations in definitions and test methods. While it is likely that some individuals entirely lack the ability to converge their eyes irrespective of training, our hypothesis is that a vast majority of the population can, after some training, converge their eyes to some extent. Only access to more data can resolve this hypothesis, where a suitable source could be the ongoing clinical study NCT06629740. However, a complicating factor is the combination of the acquired skill and the benefit of individualization, leading to the question, “When has a subject reached their typical ability to converge their eyes?” If individualization is conducted too early, it will not be representative of the individual's ultimate capacity to converge their eyes. Here, the baseline visit on the day of the intervention (V2base) was assumed to represent the ability to converge their eyes at least on the day of intervention and was hence used for individualization purposes. Even after individualization, there was variation in the baseline tests, indicating that the test method may benefit from standardization, such as requesting the subject to hold their index finger on their nose and visually focus on the finger to guide their eyes.

For 2 of the 12 subjects, the reduction in ability to converge their eyes due to lorazepam administration was small, leading to a high fraction of false negatives and false positives for these two. Of these two subjects, E demonstrated a good ability to converge their eyes both before and after BZD administration, whereas K exhibited a poor ability to converge their eyes both before and after BZD administration. Therefore, any attempt to indicate benzodiazepine use based on individualized data also needs to first confirm that an individual can converge their eyes before using this method, but this will not prevent all false negative results. Overall, this indicates that not every individual will show detectable changes in eye movement behavior after intake of 2 mg of lorazepam. However, the average performance of the eye test classifier using individualized NCdiffInd was on par with or even better than many biochemical tests (AUC = ∼0.9), with a true positive rate of ∼0.6 and a false negative rate of ∼0.05 (Figure 3). Since this classifier requires individualization, it will be apparent if an individual cannot converge their eyes even after training. By including an eligibility test based on the results from the individualization procedure, the test performance will increase because an individual incapable of converging their eyes sufficiently would be excluded upfront. Such an eligibility test would, in a real-life scenario, exclude subject K from using the test to monitor BZD use, because K failed to converge their eyes more than the classification limit (NCdiff = 0.15) when sober.

A potential use case for a widespread measurement system that indicates benzodiazepine use is to support the monitoring of a patient with substance use disorder in achieving and maintaining sobriety. Such support tools exist for other substances, for example, alcohol (31–33). For use in a therapy support situation with intensive outpatient care, test frequency is as important as test functionality (18). The time window after intake of 2 mg of lorazepam when the eye test can indicate the drug is up to 3–5 h. Hence, conducting three to four tests per day appears to be an appropriate interval for indicating drug use, which is clearly possible with a distributed smartphone app solution. Considering that drug administration in abuse situations is typically 3–10 times higher than the dose used in the clinical study (34, 35), the detection window for lorazepam is probably longer. Other benzodiazepines have variations in potency and formulation, meaning that dosing, pharmacokinetic, and pharmacodynamic profiles all impact the detection window and consequently the number of necessary measurements per day. One should also note that the smartphone-based tests were performed frequently and thus increased the potential to detect drug use compared with a single chemical test performed with 1–2-week intervals.

The smartphone solution evaluated here was built on a technology framework that has been deployed to support therapy for alcohol use disorder (31–33) for almost a decade. All the peripheral functions supporting patients in outpatient care, such as maintaining a diary, questionnaires on mood and motivation, communication, and the like, are hence readily available. The eHealth solution for alcohol use disorder patients has been shown to be helpful for patients to maintain motivation to stay sober and avoid relapses (36). The smartphone-based eye-scanning approach described in this report was recently deployed on a pilot scale, where the data collected from early experiences (37) indicates that the same could be true for patients with substance use disorder. With fewer persons misusing or abusing drugs, including BZDs, fewer children will have intoxicated parents and fewer motor vehicles will be driven under the influence, to mention two examples.

The raw NCdiff data serve as a benchmark for expected outcomes in a control scenario where data individualization is not feasible. By “control scenario,” we refer to the monitoring of BZD use in an entrance gate test situation, i.e., use outside a therapeutic context and without access to individualization. The receiver operator characteristic AUC of the classifier with native NCdiff data was 0.79, which is often categorized as within an “acceptable” range (0.7–0.8) for diagnosis. These findings derive from a small sample of 12 subjects who conducted repeated drug-free tests over the course of a week (*n* = 30–52 measurements), compared to tests conducted within 5 h of drug intake (*n* = 16–37 measurements), indicating a somewhat unbalanced logistic regression model. Despite this imbalance, with a TN rate at 90% and a TP rate at 56%, the classifier based on NCdiff alone approached a performance that can be of use in a control scenario. The performance in a control scenario could be further improved by including nystagmus data in the regression model, and since BZD exhibits its action through the GABA receptor system, it is probable that other intoxicating substances acting on the same receptor system [such as alcohol (38)] would cause a similar effect on eye function.

The strength of this study lies in the large number of tests conducted both under controlled laboratory conditions and in the at-home setting, providing a solid statistical basis for detecting differences. A limitation is that it only involved 12 healthy volunteer subjects, which introduces the risk of overestimating classifier performance. Furthermore, for ethical reasons, the administered dose was in the therapeutic range, which is often lower than the doses used in abuse situations. Possible follow-up activities include evaluating other substances that act on the same GABA receptor system and increasing the number of subjects.

In conclusion, individualized data from the eye non-convergence paradigm demonstrates an ability to indicate lorazepam use during a window of up to 3–5 h after drug intake, with tests also conducted in ambient at-home situations. Critical observations from this study are the importance of individualizing the NCdiff signal for each individual and the acquired ability to converge one’s eyes. The development of a smartphone application to monitor eye convergence represents a significant step forward in the non-invasive surveillance of benzodiazepine ingestion using consumer-grade hardware and a rapid, distributed test setting.

## Data Availability

The original contributions presented in the study are included in the article/Supplementary Material, further inquiries can be directed to the corresponding author.
